# Modeling Environmentally-Induced Motor Neuron Degeneration in Zebrafish

**DOI:** 10.1038/s41598-018-23018-w

**Published:** 2018-03-20

**Authors:** Jessica R. Morrice, Cheryl Y. Gregory-Evans, Christopher A. Shaw

**Affiliations:** 1 Experimental Medicine Program, University of British Columbia, Vancouver, Canada; 2 Graduate Program in Neuroscience, University of British Columbia, Vancouver, Canada; 3 Department of Ophthalmology and Visual Sciences, University of British Columbia, Vancouver, Canada

## Abstract

Zebrafish have been used to investigate motor neuron degeneration, including as a model system to examine the pathogenesis of amyotrophic lateral sclerosis (ALS). The use of zebrafish for this purpose has some advantages over other *in vivo* model systems. In the current paper, we show that bisphenol A (BPA) exposure in zebrafish embryos results in motor neuron degeneration with affected motor function, reduced motor axon length and branching, reduced neuromuscular junction integrity, motor neuron cell death and the presence of activated microglia. In zebrafish, motor axon length is the conventional method for estimating motor neuron degeneration, yet this measurement has not been confirmed as a valid surrogate marker. We also show that reduced motor axon length as measured from the sagittal plane is correlated with increased motor neuron cell death. Our preliminary timeline studies suggest that axonopathy precedes motor cell death. This outcome may have implications for early phase treatments of motor neuron degeneration.

## Introduction

The majority what we currently understand about the pathogenic mechanisms driving motor neuron degeneration, particularly in amyotrophic lateral sclerosis (ALS) has been based on genetic models of disease^1,2^. Zebrafish offer unique advantages for modeling aspects of some motor neuron diseases, including in ALS. Zebrafish have a functionally and anatomically similar, yet simplified nervous system compared to humans^3–5^ and genetic mutations implicated in neurodegenerative diseases are often highly conserved^5,6^. Adult zebrafish have a high fecundity, where the embryo develops *ex vivo*^7^ and both adult and embryonic zebrafish have been used extensively for toxin screening studies^4^. In context to modeling ALS in embryos, zebrafish offer a convenient method for estimating motor axon abnormalities. The motor system rapidly develops which can be directly observed *in vivo*. The conventional method to estimate motor neuron degeneration in embryos is analysis of caudal primary (CaP) motor axon length as these motor neurons have the most prominent axonal projection^8–12^. Further, embryos have simple stereotypical motor behaviours which are useful for investigating affected motor function^13^.

ALS is characterized by the progressive degeneration of both upper and lower motor neurons^1,14^. There are two main forms of ALS with about 90% of cases considered to be of sporadic origin, termed sALS. These cases lack evidence of a hereditary genetic component. The remaining 10% of cases are familial ALS (fALS) and appear to arise from different genetic mutations^15^. Although sALS and fALS patients have a similar clinical pathology at end-stage disease^16^, it is currently unknown if both forms result from a similar pathogenic mechanisms and pathways, and further how relevant research based on genetic models is to sALS pathogenesis^17^.

Epidemiological studies have suggested that an array of different ALS “risk factors” are associated with sALS cases^18–26^, including different neurotoxins such as heavy metals, pesticides, insecticides^21^ and diet (*β*-sitosterol *β*-d-glucoside “BSSG”)^23^. More recently, endocrine disruptors have also been implicated in neurodegenerative diseases^27,28^ such as ALS^29^. Despite evidence for various environmental factors having a role in disease etiology, a causal relationship in humans has yet to be established^17^.

In the present article, we build upon previous findings^30^ of motor defects in zebrafish exposed to the well-characterized endocrine disruptor bisphenol A (BPA). We use this toxin, not to link it specifically to ALS, but rather as a means to examine the general processes which may underlie motor neuron degeneration. Although BPA has not previously been associated with motor neuron disease, the molecular cascade leading to motor neuron death may be similar across various environmental toxins, many of which may be key factors in sALS. Further, we demonstrate that the use of reduced motor axon length is a valid surrogate marker of motor neuron degeneration and provide preliminary evidence to suggest retrograde motor neuron degeneration in this model of toxin-induced motor neuron pathology.

## Results

### Dose-dependent effect of BPA exposure on mortality

We initially investigated the dose-dependent effect of BPA on mortality and motor axon structure. Mortality was assessed at 24 and 48 hpf (Table 1). At 24 hpf, no increased mortality was observed in any exposure dose. At 48 hpf, increased embryonic mortality was evident in the higher doses, where 100% mortality resulted from exposure to 100 *μ*M BPA.

**Table 1:** Lethal exposure doses. Percent mortality of wild type embryos at 24 hpf and 48 hpf. N = 14**–**15 biological replicates.

	Control^Ψ^	Vehicle control*	15 μM BPA	50 μM BPA	60 μM BPA	70 μM BPA	80 μM BPA	90 μM BPA	100 μM BPA	200 μM BPA
24 hpf	0%	0%	0%	0%	0%	0%	0%	0%	0%	100%
48 hpf	0%	0%	0%	0%	6.7%	0%	0%	26.7%	100%	NA

Embryos were subject to chronic non-static exposure of treatment starting at 6 hpf in all groups.

^Ψ^E3 media.

^*^1% DMSO dissolved in E3 media.

NA: this dose was 100% lethal to embryos at 24 hpf.

Abbreviations: BPA – Bisphenol A; hpf – hours post fertilization.

### Effect of BPA exposure on motor function

Motor behaviour in zebrafish embryos was investigated to determine the effect of BPA exposure on motor function. We observed that wild type embryos exposed to 50 μM BPA show reduced motor behaviour as indicated by the touch evoked escape response (TEER) assay at 24 hpf and 48 hpf (86.7% and 100% respectively) compared to control and vehicle control embryos (Table 2).

**Table 2:** Motor function. Percent of wild type embryos with reduced motor function at 24 hpf and 48 hpf using TEER. N = 15 biological replicates; N = 1 technical replicate.

	Control^Ψ^	Vehicle Control*	50 μM BPA
24 hpf	6.7%	0%	86.7%
48 hpf	0%	0%	100%

Embryos were subject to chronic non-static exposure of treatment starting at 6 hpf in all groups.

^Ψ^E3 media.

*1% DMSO dissolved in E3 media.

Abbreviations: BPA – Bisphenol A; hpf – hours post fertilization.

### Dose-dependent effect of BPA exposure on motor axon length and branching

**Figure 1:**
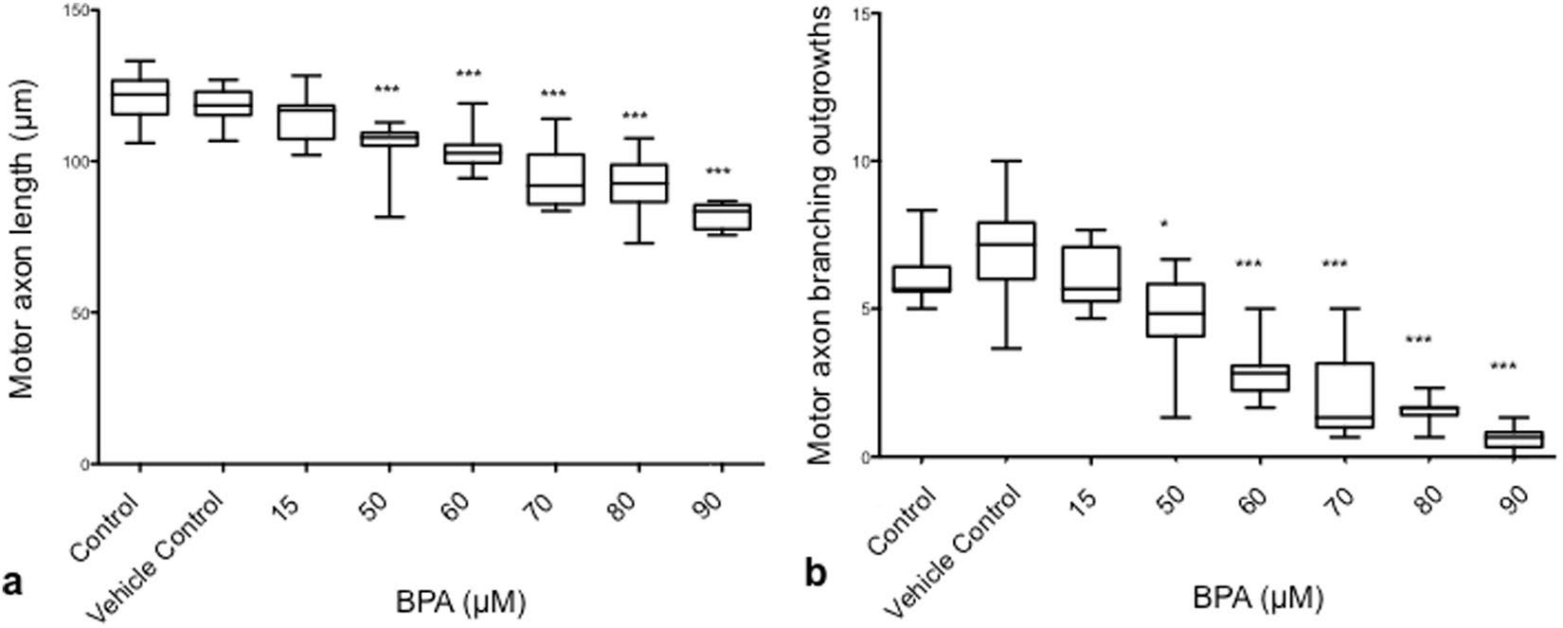
Dose-dependent effect of BPA on motor axon length and branching on wild type embryos. (**a**) Motor axon length under increasing concentrations of BPA. (**b**) Total number of motor axon branching outgrowths under increasing concentrations of BPA. *P = 0.034, ***P < 0.001). Embryos were subject to 42 hours duration of exposure to different concentrations of BPA. Data are based on the mean value of N = 5–6 technical replicates (motor axon length), N = 2 technical replicates (motor axon branching) and N = 8–10 biological replicates. P values were determined by Kruskal-Wallis ranked sums and Mann-Whitney U post test.

To understand the effect of BPA on motor axons, we characterized the effect of increasing exposure doses on motor axon length and branching. Exposure to 50 μM BPA significantly reduced both motor axon length (P < 0.0001, N = 10 biological replicates) and branching (P = 0.034, N = 10 biological replicates) from control values at 48 hpf (Fig. 1a,b). This effect on reduced motor axon length and branching was dose-dependent with increasing doses of 60–90 μM BPA (Fig. 1; P < 0.001, N = 8–10 biological replicates). We also found that BPA has a duration of exposure effect on motor axon length, where no significant effect on reduced motor axon length was observed at 24 hpf (Supplementary Fig. S1). From these data, the optimal BPA exposure dose on both reduced motor axon length and branching without increased mortality was determined to be 50 *μ*M, this dose was used for all subsequent experiments.

### Neurodevelopmental exposure of BPA on adverse motor effects

To determine if the neurotoxic effect of BPA is based on the neurodevelopmental time point of exposure, embryos were exposed to BPA at two different developmental points instead of 6 hpf: 12 hpf and 3 days post fertilization (dpf). Embryos exposed to BPA starting at 12 hpf were analyzed at 24 hpf and 48 hpf, and embryos exposed to BPA at 3 dpf were analyzed prior to any exposure at 3 dpf and post-exposure at 5 dpf. Embryos exposed to BPA at 3 dpf followed the equivalent 42 hour duration of exposure as experiments in Fig. 1, Table 2.

Delayed developmental exposure to BPA at 12 hpf had no effect on mortality at 24 hpf or 48 hpf compared to vehicle controls (Supplementary Table S1). At 24 hpf, BPA exposed embryos did not show abnormal motor function as evidenced by TEER. At 48 hpf, reduced motor behaviour was evident in 100% of BPA exposed embryos as indicated by no observed escape response using TEER (Supplementary Table S2). Our results show that delayed neurodevelopmental exposure to BPA at 12 hpf caused significantly reduced motor axon length at 48 hpf as compared to vehicle controls (Supplementary Fig. S2; P = 0.04, N = 8 biological replicates). We note that embryos subject to this delayed BPA exposure had 6 hours less exposure duration than embryos exposed to BPA at 6 hpf as analyzed at 48 hpf.

**Figure 2:**
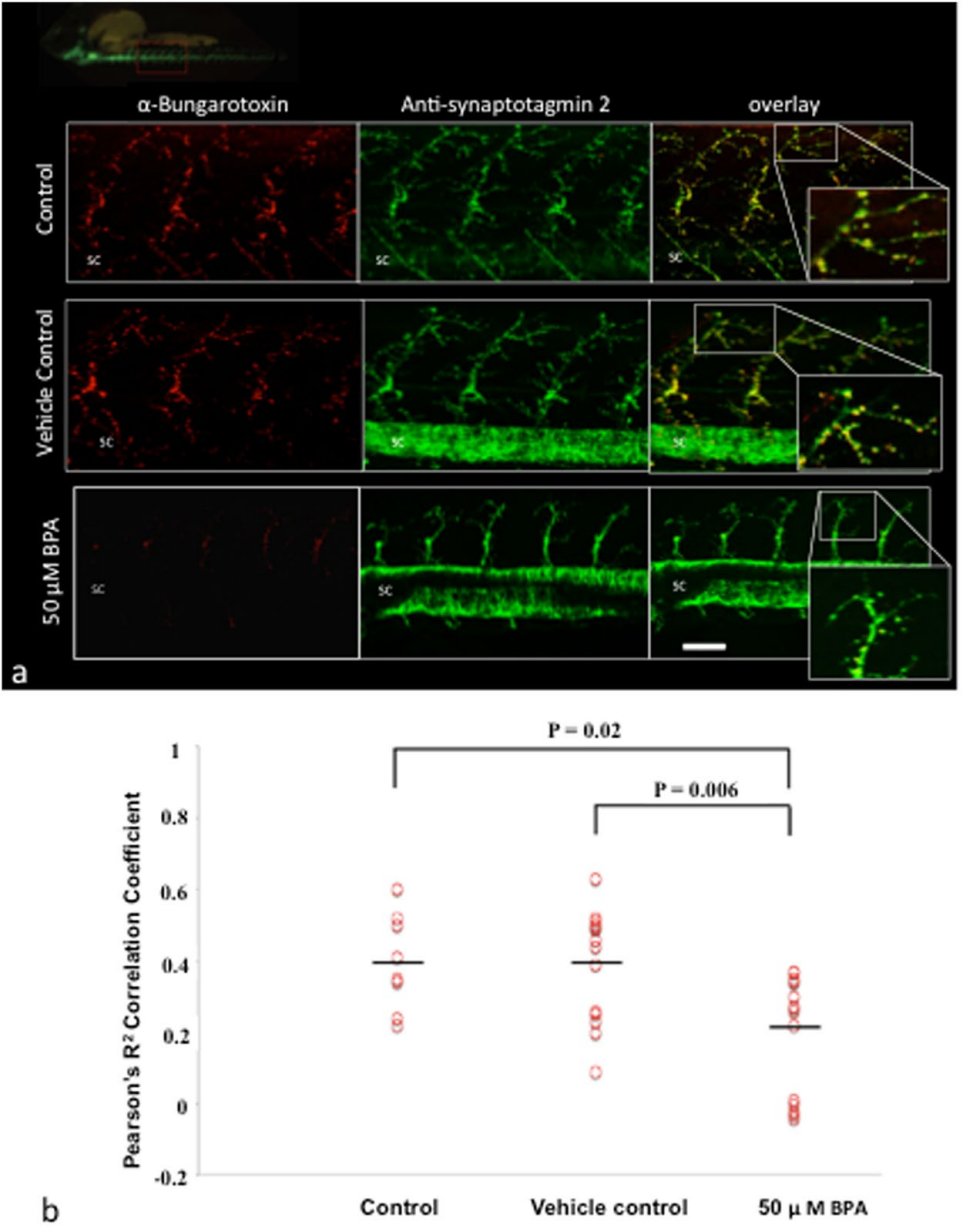
BPA exposure impairs NMJ integrity. (**a**) Representative images of α-bungarotoxin (post-synaptic terminal) and synpatotagmin 2 (pre-synaptic terminal) labeling in wild type embryos at 48 hpf exposed to BPA vehicle control, or control media. Inset magnifications are merged images of the pre- and post-synaptic terminals. SC, spinal cord. (**b**) Data are based on the integrity of the NMJ at 48 hpf in control, vehicle control and BPA exposed embryos as measured by Pearson’s R^2^ co-localization coefficient between α-bungarotoxin and synaptotagmin 2. P = 0.02, N = 8–15 biological replicates; P = 0.006, N = 15 biological replicates; N = 5–6 technical replicates of motor neurons. P values were determined by Mann-Whitney U test.

**Figure 3:**
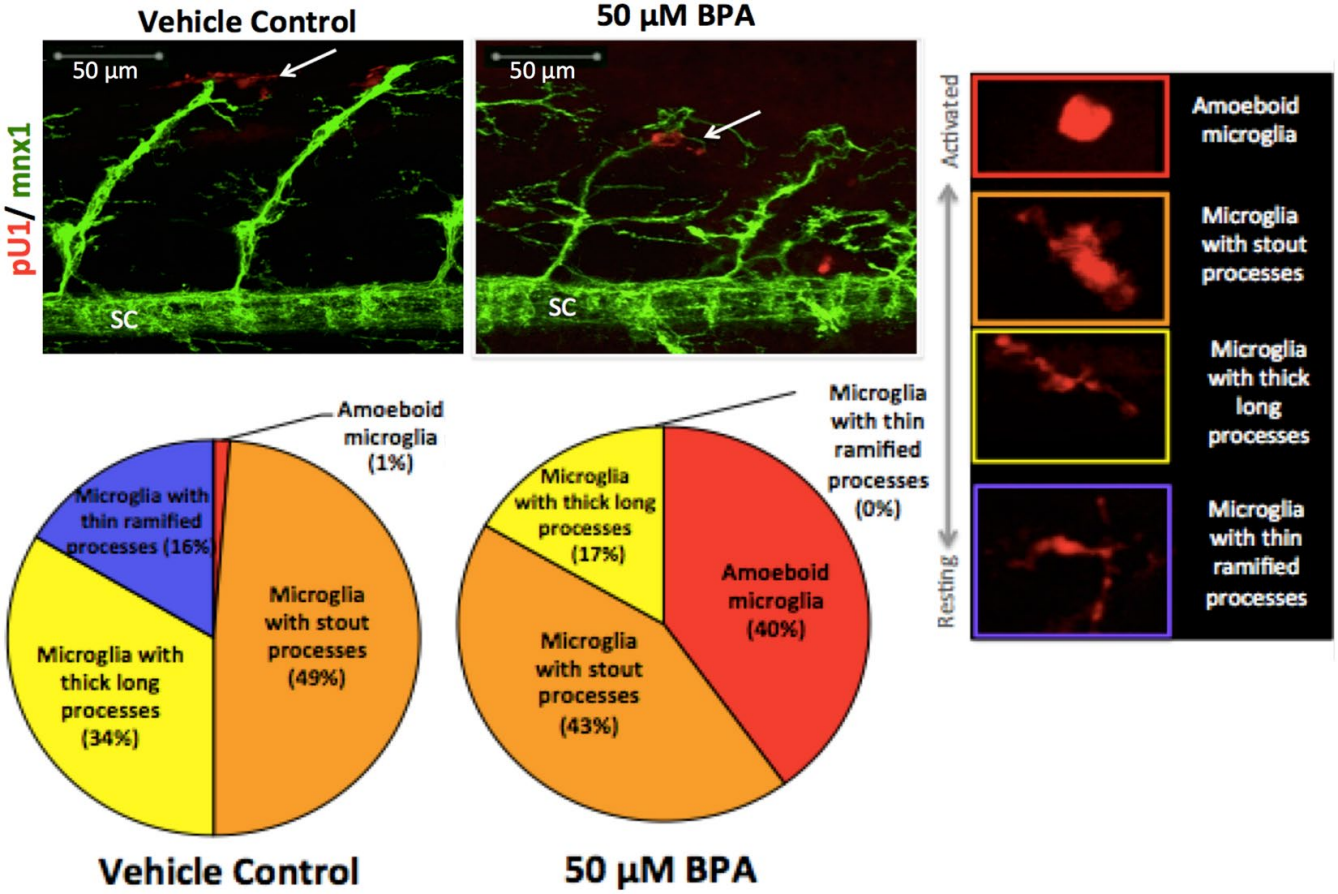
Activated microglia associate with defective motor neurons. Double transgenic *Tg:mnx1-GFP/pU1-RFP* embryos identify microglia (red) and motor neurons (green). White arrows point to pU1+ microglial cells spatially associated with motor neurons at 48 hpf. Percent values in the pie charts show the relative proportion of total microglia in each activation state compared to total number of microglia cells in region of interest (right panel) when exposed to BPA compared to vehicle controls (N = 9–12 biological replicates; N = 1–10 technical replicates). SC = spinal cord.

**Figure 4:**
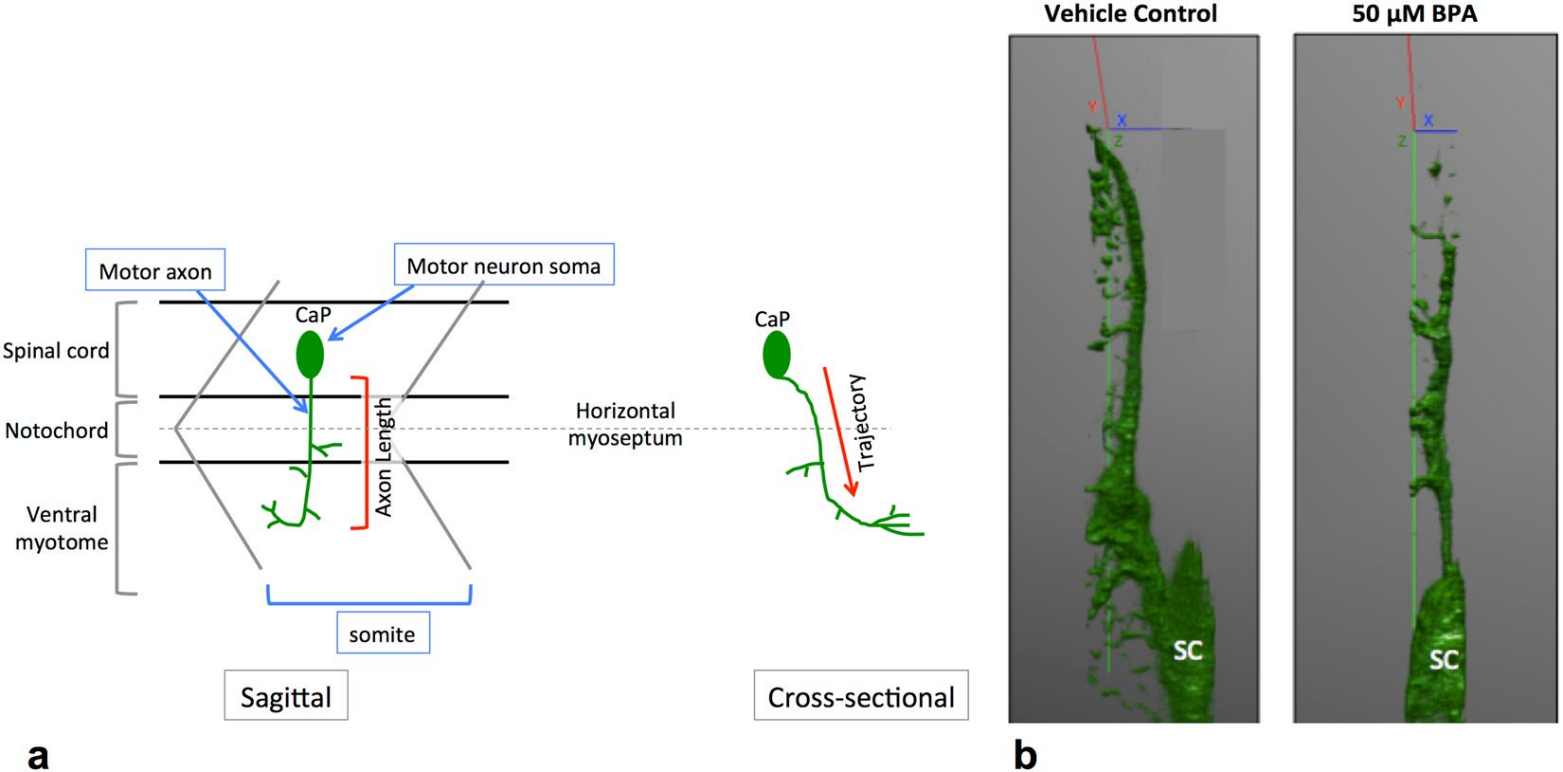
Motor neuron trajectory from a sagittal and cross sectional view. At 18 hpf, primary motor neurons start to exit the ventral root of the spinal cord. By 48 hpf each somite has one set of caudal (CaP), middle (MiP) and rostral (RoP) primary motor neurons innervating each side of the spinal segment^67^. For simplicity, here we only show the CaP motor neuron. (**a**) CaP motor axons project ventrally from the SC to the ventral myotome. Axon length is conventionally measured from the sagittal plane, with the assumption that motor axons in control embryos and embryos with motor axon abnormalities follow the same trajectory. (**b**) Defective motor axons have a normal trajectory from the spinal cord. *Tg:mnx1-GFP* embryos exposed to BPA have a similar motor axonal trajectory through the Z axis (green) as vehicle controls (N = 3 biological replicates).

To investigate if BPA is causing an abnormal motor phenotype by affecting the development of motor neurons, BPA was exposed to embryos at a later developmental time point of 3 dpf for 42 hours of duration, which is an equivalent exposure duration as used in other experiments (Figs 1–4. Prior to any treatment exposure, the motor behaviour of embryos was analyzed at 3 dpf to estimate basal motor behaviour and 93.3% of embryos showed normal motor behaviour with an observed escape response (Supplementary Table S3). Embryos exposed to BPA starting at 3 dpf showed 3.3% mortality at 4 dpf and 93.7% mortality at 5 dpf (Supplementary Table S4), suggesting that embryos are more sensitive to the toxic affects of BPA, or are less tolerant of BPA exposure in later development. At 5 dpf, reduced motor behaviour was observed in 100% of the remaining BPA-exposed embryos (Supplementary Table S3). Reduced motor axon length at 5 dpf was evident in BPA-exposed embryos as compared to vehicle controls (Supplementary Fig. S3; P = 0.03, N = 8 biological replicates). Together, these findings indicate that the neurotoxic effect of BPA on the motor phenotype does not depend on neurodevelopmental time point of exposure, and suggest that embryos in earlier stages of development are more tolerant to the toxicity of BPA.

### Neuromuscular junction (NMJ) integrity

The NMJ integrity was analyzed to further investigate our finding of BPA-induced motor axon abnormalities (Fig. 1) and affected motor behaviour (Table 2). Analysis of colocalization between pre-synaptic (synpatotagmin 2) and post-synaptic (*α*-Bungarotoxin) terminals show that embryos exposed to 50 *μ*M BPA had significantly reduced NMJ integrity at 48 hpf compared to both vehicle control groups (Fig. 2; P = 0.006, N = 15 biological replicates) and controls (P = 0.02, N = 8 biological replicates).

### Microglia activation

To investigate microglia activation following BPA exposure, microglia cells (pU1+ cells) spatially associated with motor axons and motor soma were graded based on morphology indicative of the activation state. Microglia in a quiescent state have a ramified morphology and once activated, adopt an amoeboid morphology^31^. At 48 hpf, embryos exposed to 50 µM BPA had a higher proportion of microglia in an activated state: 40% amoeboid microglia, 0% microglia with thin ramified processes, whereas the vehicle control group had a higher proportion of microglia in a quiescent state: 1% amoeboid microglia, 16% microglia with thin ramified processes (Fig. 3).

**Figure 5:**
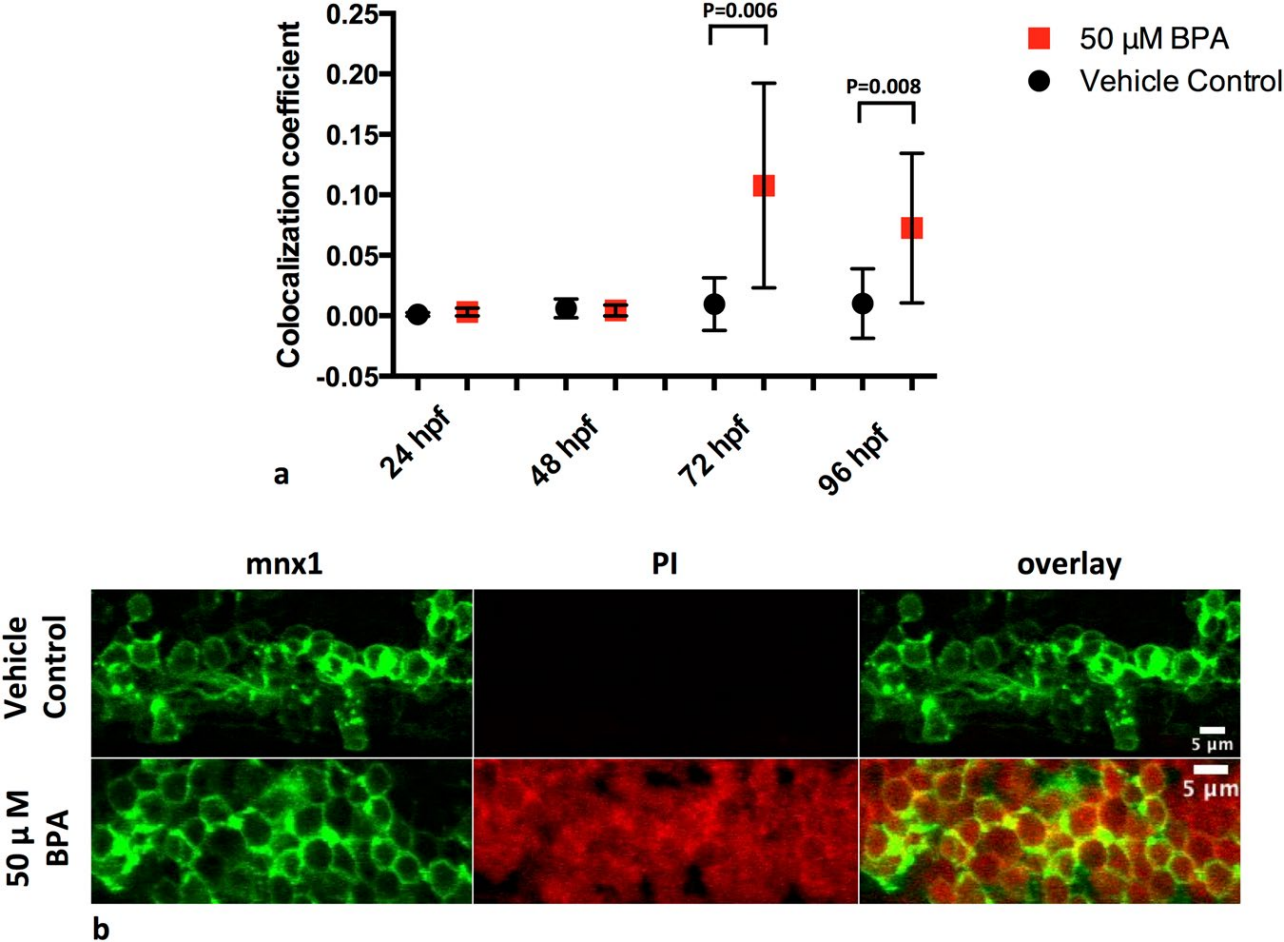
BPA exposure causes increased motor cell death at 72 hpf. (**a**) Motor neuron cell death is increased in the spinal cord starting at 72 hpf following BPA exposure as compared to vehicle controls. Values indicate colocalization coefficients between cells with a positive signal for both mnx1-GFP and PI in the spinal cord, indicating dead motor neurons. Group values between time points should be interpreted independently due to non-uniform PI staining between time points. (**b**) BPA-exposed embryos show colocalization between PI + cells and mnx1 motor neuron soma 96 hpf, and show that PI staining is not specific to mnx1 motor neuron soma. Data represent the mean ± s.d.; N = 7–12 biological replicates. P values were determined by Mann-Whitney U test.

Our results show that motor axon length and motor behaviour were significantly reduced at 48 hpf (Fig. 1, Table 2) and motor neurons undergo cell death starting at 72 hpf (Fig. 5). To investigate the role of microglia during BPA-induced motor neuron degeneration, we analyzed microglial spatial localization and phagocytosis during later stages of pathogenesis. A time point analysis of microglia during two stages of motor neuron degeneration show that activated microglia associated with degenerating motor axons and not the motor neuron soma at 48 hpf (Supplementary Table S5, Video S1) and engulf apoptotic neurons in the spinal cord at 72 hpf (Supplementary Fig. S5).

### Motor axon length as a surrogate marker of neurodegeneration

To determine if the 2-dimensional motor axon length measured from the sagittal plane accurately represents the length of the 3-dimensional axonal trajectory through the Z axis, or if the angle of trajectory is such that a 2-dimensional view would misrepresent the true length (Fig. 4a), we evaluated the deviation of the motor axon trajectory of environmentally-induced motor axon abnormalities as viewed from the cross sectional plane. BPA exposed embryos with reduced motor axon length from a sagittal view had a similar cross-sectional trajectory through the Z axis as compared to vehicle controls at 48 hpf (Fig. 4b).

Further, to confirm that reduced motor axon length and branching are correlated with motor neuron cell death and to determine if BPA induces motor neuron death, we investigated evidence for motor neuron cell death. Different mechanisms of cell death have been implicated in ALS^32^. Therefore to investigate motor neuron cell death, propidium iodide (PI) was used as a non-pathway specific cell death marker. Chronic, non-static exposure to BPA resulted in increased embryonic mortality starting at 96 hpf (Table 3). Colocalization between GFP+/PI+ cells in the spinal cord showed evidence that embryos exposed to 50 *μ*M BPA had significantly increased motor cell death at 72 hpf and 96 hpf (Fig. 5; P = 0.006, N = 9–11 biological replicates and P = 0.008, N = 9–10 biological replicates, respectively) as compared to vehicle controls, although we note that BPA induced cell death was not specific to motor neurons (Supplementary Fig. S7).

**Table 3:** Lethal exposure duration. Duration of BPA exposure on embryonic mortality from 24 hpf to 120 hpf in *Tg:mnx1-GFP* embryos (% mortality of total embryos). N = 8–33 biological replicates.

	24 hpf	48 hpf	72 hpf	96 hpf	120 hpf
Vehicle Control*	3.9%	0%	1.6%	0%	0%
50 μM BPA	1.9%	2.0%	5.7%	40%	100%

Embryos were subject to chronic non-static exposure of treatment starting at 6 hpf in all groups.

*1% DMSO dissolved in E3 media.

Embryos were subject to chronic non-static exposure of treatment starting at 6 hpf in all groups for the duration of embryonic development indicated.

Abbreviations: BPA – Bisphenol A; hpf – hours post fertilization.

To determine if motor neuron degeneration is being driven by muscle damage, or a myopathy rather than motor neuron degeneration, we tested the birefringence of skeletal muscles, which assess muscle integrity^33^. We found that BPA exposed embryos had comparable birefringence of the trunk skeletal muscles to vehicle controls at 48 hpf (Supplementary Fig. S6).

## Discussion

In the present study, we modeled toxin-induced motor neuron degeneration by exposing zebrafish embryos to BPA. Our results demonstrate that BPA exposure results in reduced motor function, abnormal motor axon structure, decreased NMJ integrity, and the presence of activated microglia. These are useful tools to further investigate the pathogenesis underlying motor neuron degeneration in zebrafish models, including those of ALS. We note, however, the current study’s limitations which include data from adult zebrafish, the inherent differences in physiological processes involved in toxin-induced neurodegeneration between humans and zebrafish, and the use of an environmental toxin lacking a previous association with ALS.

Here, we build upon previous research^30^ demonstrating BPA’s effect on the motor system in zebrafish. To investigate BPA exposure as a method to environmentally-induce motor neuron degeneration, we focused on quantifying BPA’s effects on the CaP motor neuron, which is a conventional method to ALS studies in zebrafish embryos. Our results did not show significantly reduced CaP motor axon length or branching at doses used by previous groups to demonstrate motor defects, therefore we use a higher dose to pursue this method as a model for motor neuron degeneration. We confirm that BPA-induced motor axon defects are correlated to motor neuron cell death in zebrafish. Further, results from this model confirm that motor axon defects are reliable markers for motor neuron degeneration and not necessarily the result of aberrant axonal growth. Future studies investigating the mechanism of action underlying BPA-induced motor neuron degeneration is warranted.

Thus, these results suggest that this model may be valuable for investigating the pathogenesis underlying environmentally-induced neurodegeneration, including that which may underlie sALS. It is important to emphasize that the focus of this research is not to claim that BPA exposure is related to ALS. Rather, the rationale behind investigating the effects of BPA on motor neuron degeneration is that the cascade of events triggered by BPA may mirror those by other toxic factors that are more likely epidemiologically valid for ALS. A very similar set of assumptions lies behind the use of genetic models of ALS, e.g., mSOD, in which the mutation is linked to only a small fraction of all ALS cases.

Environmental factors are largely believed to be involved in sALS etiology^18–22^, although to date the type of agents involved and their underlying pathogenic mechanisms are not well understood. There is currently no direct evidence that BPA exposure is involved in ALS pathogenesis, yet motor neuron degeneration caused by environmental stressors may follow similar mechanisms and thus may be representative of sALS pathogenesis. BPA is a well characterized endocrine disruptor to which humans are ubiquitously exposed^34^. It is known to either activate or antagonize many different receptors such as estrogen receptors (ERs) *α* and *β*^35^, membrane-bound estrogen receptor GPR30, glucocorticoid receptors and androgen receptors (AR)^34^. BPA is structurally similar to 17*β*-Estradiol (E2), the most prevalent estrogen in humans. As a xenoestrogen, BPA is known to bind to ERs and elicit weak estrogenic properties^34,36,37^. It has been established that E2 has a neuroprotective effect on in spinal motor neurons^38,39^ and has been proposed as a potential therapeutic for ALS patients^38,40–42^. The mechanism of neuroprotection is believed to function independently of ER-mediated mechanisms^38,39^, and it is currently unclear whether antioxidant activity^38,39^ or the GPR30^38,43^ is promoting cell survival.

Endocrine disruptors can be implicated in neurodegeneration by altering hormone receptors^29,44^, free radical generation^27^, neurotransmitter impairment^28^, or by mimicking the hormones that are, or may be associated with particular diseases^44^. Estrogen is critical to the development and health of the nervous system^16^. To our knowledge, there is currently no epidemiological evidence to suggest an association between BPA and ALS. The mechanism of action underlying BPA’s effects on motor neurons is currently unclear, although it’s estrogenic endocrine disrupting properties remain possible. ALS has been associated with heavy metals that have endocrine disrupting qualities^29,45,46^. For example, metals associated with ALS such as cadmium^47^ and manganese^48^ have been shown to have an effect on estrogenic activity^16^. However, a similar dose of BPA as used by our group has been reported to induce apoptosis mainly in the cells of the brain and spinal cord in Xenopus laevis embryos, which did not appear to be due to BPA’s estrogenic properties^49^. Here, we observed that BPA exposure shows evidence of a time-dependent exposure effect on motor neuron toxicity and these effects did not depend on neurodevelopmental stage of exposure. It is currently unclear how motor neuron degeneration is being driven in this model, which warrants further investigation. It remains possible that BPA may elicit effects on motor neurons through non-androgenic receptor-independent pathways^34^, hydroxyl radical formation^27^ or through pathways involving estrogen-responsive nuclear respiratory factor 1 (NRF1) target genes^29^.

In this study, we found that BPA has a dose-dependent effect on reduced motor axon length and branching, features often used as surrogate markers of motor neuron degeneration in zebrafish studies of motor neuron degeneration. It is important to ensure that motor neurons with reduced axon length and branching are indeed undergoing cell death in order to confirm that these motor neurons are undergoing degeneration, not simply aberrant growth. Our data show evidence of increased motor neuron cell death, although we note that BPA-induced cell death was not specific to motor neurons. To further validate this method, we found evidence that the CaP motor axon length as measured from a 2-dimensional sagittal plane at 48 hpf is an effective surrogate marker of motor neuron cell death and is also representative of the motor axon trajectory through the cross-sectional plane. This model thus provides evidence to confirm the conventional method used in embryonic zebrafish studies of motor neuron degeneration which have previously relied on reduced motor axon length as a marker of motor neuron degeneration^8–11^.

Microglia are now understood to have an operative role during motor neuron disease, including in ALS pathogenesis, where pronounced activation is evident in both ALS patients and in disease models^50,51^. Microglia are found in a neurotrophic state during early disease pathogenesis and are associated with an anti-inflammatory profile which progresses to a more pro-inflammatory, neurotoxic phenotype during later stages of pathogenesis^52^. The microglial phenotype is directly affected by their specific microenvironment and removing microglia from their native milieu can skew the phenotype, a fact which should be considered when interpreting microglia *in vitro* studies^31^. In the current study, we found evidence of microglia activation in cells that are spatially associated with motor axons during early stages of motor neuron degeneration. To confirm sustained microglial activation, techniques such as live imaging to trace these cells is necessary.

In ALS, there is evidence for both anterograde “dying forward”^53^ and retrograde “dying back”^54^ motor neuron degeneration. It is currently unknown where motor neuron cellular dysfunction initiates during the degenerative process or if both retrograde and anterograde degeneration occur concurrently^55^. In this study, we investigated a time point analysis of motor neuron degeneration and found that motor axon abnormalities were evident at 48 hpf and that increased motor neuron cell death was observed starting at 72 hpf. These data indicate that motor neuron dysfunction precedes motor cell death, suggesting that in some cases, motor neuron pathogenesis initiates at the motor axon, not the soma. We note that further studies are required to confirm this finding. We did not find evidence of a myopathy at 48 hpf, which supports that the motor defects reported in this research are demonstrative of motor neuron degeneration instead of being caused by muscle damage. Further, we show that at 48 hpf, activated microglia are associated with motor axons, but not the soma. Our live imaging data show that microglia engulf apoptotic neurons in the spinal cord at 72 hpf. These data suggest that toxin-induced motor neuron may occur by retrograde degeneration, although further studies are needed to confirm this. Similar conclusions, as cited above, have been reached by other investigators using the mSOD model of fALS^54^.

In conclusion, we show that BPA-induced neurotoxicity recapitulates certain hallmarks of motor neuron degeneration in zebrafish and our results are suggestive of motor axonopathy as the site of upstream pathogenesis. Further, we show that the conventional method of estimating motor neuron degeneration using the length of motor axons is a valid measure of motor neuron cell death. Together, these findings demonstrate that zebrafish are valuable to model toxin-induced motor neuron degeneration and may provide valuable insight into the pathogenesis underlying toxin-based neurodegenerative diseases, such as sALS. Studies investigating the underlying pathogenesis driving BPA’s effect on motor neurons and the site of initial dysfunction are warranted. Our present results based on modeling a degenerative motor neuron phenotype as conventional in zebrafish studies of ALS and exposure doses are higher exposure doses than is environmentally relevant^56^. Investigating a lower concentration is worthwhile for future studies which could focus on BPA or endocrine disruptors involvement in motor neuron degeneration. Future studies modeling sALS in zebrafish could focus on environmental stressors previously associated with ALS to perform high throughput screening of neurotoxin-induced motor neuron degeneration. This may be valuable to further investigate the upstream pathogenic mechanisms driving neurotoxin-induced motor neuron degeneration.

## Methods

### Fish husbandry and lines

Zebrafish (*Danio rerio*) were bred and maintained according to standard procedures^57^. All experiments listed were performed within the relevant guidelines and regulations of the Canadian Council for Animal Care (CCAC) and the experimental protocols were approved by the local Animal Care Committee of the University of British Columbia. We use the wild type AB and the transgenic *Tg*:*mnx1-GFP*^58^ (ZFIN ID: ZDB-ALT-051025-4) and *Tg:pU1::Gal4-UAS::TagRFP*^3^ zebrafish strains for all experiments listed. Embryos were raised at 28.5 °C on a 14 h light/10 h dark cycle in 100 mm^2^ petri dishes containing aquaria water. To inhibit pigment formation in embryos and produce clear images using confocal microscopy, 0.003% phenylthiourea (PTU) (v/v) was added to the embryo treatment media.

### Bisphenol A (BPA) exposure

A stock solution of 10 mg mL^−1^ BPA was prepared by dissolving BPA granules (Sigma-Aldrich) in 100% DMSO using a vortex. Aliquots were stored at −20 °C and replaced every 6 months with freshly prepared stock solution. Final working solutions were prepared by dissolving BPA solution in E3 media (5 mM NaCl, 0.17 mM KCl, 0.33 mM CaCl_2_, 0.33 mM MgCl_2_, 1% methylene blue (v/v)), with a maximum final volume of 1% DMSO.

Embryos were developmentally staged at 5 hours post-fertilization (hpf) and only those with normal morphology were selected for analysis. Approximately 12 embryos per well were placed in a 6 well plate with 5 mL treatment or control media. At 6 hpf, embryos were dechorionated using 1 mg mL^−1^ pronase. Embryos were then immediately exposed to various concentrations of BPA. Groups treated with vehicle control media were administered 1% DMSO dissolved in E3 media. Groups treated with control media were administered E3 media alone. Mortality was assessed by observing the lack of a heartbeat for 10 seconds. Dead embryos were removed at least once per day. To reduce any confounding toxicity effect of static media exposure, freshly prepared BPA-treated media was administered once per day until sacrifice. Embryos lacking gross structural malformation of the head and trunk were selected for further analysis. Embryos were over-anaesthetized in tricaine, then fixed in 4% PFA and placed in 1 × PBS at 4 °C. The difference between biological replicates in each experiment was due to the loss of embryos from such as things as mortality, during dechorionation, post-treatment screening for transgenesis and double transgenesis and during washing steps. Further, some embryos are damaged during yolk sac removal for flat mounting, or are positioned improperly for imaging during the mounting process.

For delayed treatment exposure, methods were followed as stated above with the exception that embryos were exposed to experimental treatments at 10 hpf or 3 days post fertilization (dpf). As embryos naturally hatched from their chorion by 3 dpf, pronase solution was not used for this experiment.

### Whole mount immunohistochemistry

For immunolabeling of motor axons, embryos were first subject to an antigen retrieval step by incubation in 150 mM Tris HCl pH 9 for 15 minutes at 70 °C, washed two times in 0.1% PBS-Tween 20 in then incubated in acetone at −20 °C for 15 minutes. Embryos were washed three times in 0.1% PBS-Tween 20 incubated then blocked for 1 hour at room temperature (RT) with 10% normal serum, 0.8% Triton X-100, 1% BSA in 0.1% PBS-Tween 20, followed by overnight incubation at 4 °C with primary antibody znp-1 (1:200, Developmental Studies Hybridoma Bank Cat# znp-1, RRID:AB_2315626) to label motor axons and branching. The primary antibody was removed from embryos by washing two times with 0.8% PBS-Triton X-100 and three times with PBS-TS (1% Triton, 10% normal serum, 1 × PBS) and embryos were then incubated in secondary antibody FITC goat anti-mouse (1:500, Jackson Laboratories cat # 115-096-006) for 2 h at RT. Embryos were washed twice in PBS-TS and stored in 1X PBS at 4 °C until flat mounting.

### Neuromuscular junction (NMJ) labeling

Embryos were permeablized using 10 *μ*g ml^−1^ proteinase K (Promega) solution. Whole mount immunohistochemistry was performed as described above using znp-1 (or anti-synaptotagmin 2) to label pre-synaptic motor axon terminals. Following the primary antibody washing steps, embryos were incubated in 10 *μ*g ml^−1^ *α*-bungarotoxin-rhodamine conjugate (Life Technologies, cat# T1175) for 30 min at RT. Embryos were then washed once with PBS-TS before the secondary antibody was applied.

### Analysis of motor axon length

Following flat mounting, motor neurons were imaged in sagittal Z-stack sections by confocal microscopy. All images were captured within the 6–10 somite region using 20 × objective lens with a Zeiss LSM 510 laser scanning confocal microscope. Maximum projection images were reconstructed from Z-stack images using the Zen 2009 software. Maximum projection images were exported as TIFF files and analyzed using the ImageJ software. First, the scale bar was calibrated in ImageJ, then the freehand trace tool was used to measure the distance where the CaP motor axons extended from the spinal cord to the most ventral axonal projection. The mean length of 5 axons per embryo (5 technical replicates) was recorded and the mean of each treatment group (biological replicates) was reported ± s.d.

### Analysis of motor axon branching

To analyze motor axon branching, TIFF files were uploaded into ImageJ. The mean number of branches >10 *μ*m emerging from the two most rostral motor axons per embryo within the 6–10 somite region were recorded and the mean ± s.d. from each treatment group was reported.

### Motor behaviour

Motor behaviour was tested using TEER. At 24 hpf and 48 hpf, the tail region was lightly stimulated using blunt forceps and embryonic movement was recorded using a Leica MZ16 F microscope. Embryos were considered to have reduced motor function when no movement was observed within 10 seconds following a light tactile stimulation on the tail region.

### Motor neuron morphology

Embryos were imaged by confocal microscopy using a 40× water emersion objective lens. LSM files containing Z-stack images were uploaded to NeuronStudio software and reconstructed in the 3D viewer. The ZY axis was set to view the motor axon trajectory through the Z plane. Analysis of motor axon trajectory was based on deviation from the Z axis. Trajectory of 3 axons (technical replicates) and 3 embryos per group (biological replicates) were qualitatively analyzed for deviation from the Z axis as compared to vehicle controls.

### Time point analysis of motor neuron cell death

*Tg:mnx1-GFP* embryos were exposed to chronic, non-static 50 *μ*M BPA or vehicle control and fixed in 4% PFA at 24 hpf, 48 hpf, 72 hpf, 96 hpf and 120 hpf. Analysis of motor neuron cell death was performed by whole mount staining with 12 *μ*M propidium iodide (PI) for 13 minutes. LSM files of Z-stack images of GFP + motor neuron soma and PI + cells in the 6–10 somite region of the spinal cord were attained by confocal microscopy using a 40× water emersion objective. The Cy3 laser had to be adjusted between time points because PI staining was not uniform. Therefore, cell death can be interpreted between the treatment group and control for each time point and not between the different time points.

### Colocalization analysis

Z-stack LSM files were uploaded to FIJI (a version of ImageJ) and the channels were split. An ROI was carefully placed over the area of interest. Colocalization analysis was performed for each Z-stack using the Coloc 2 plugin, and Costes threshold was applied.

### Microglia phenotype

Microglia phenotype was determined based on morphology of RFP-labeled pU1+ cells in the spinal cord and surrounding motor axons. *Tg:mnx1-GFP/pU1-RFP* double transgenic embryos were fixed at 48 hpf in 4% PFA. Imaged were collected using a 20× objective lens by confocal microscopy within the 6–10 somite region. Microglia markers, origin, as well as their morphological responses to injury are largely conserved between mammals and zebrafish^59–64^. Therefore, the morphology of microglia was graded based on previously published criteria based in rats^65^. A pU1+ cell was graded if its complete structure was included in the region of interest. Due to mosaic expression of the Gal4-UAS reporter system^66^, we have reported the proportion of RFP-expressing pU1+ microglia cells instead of discrete counts. The proportion of number of microglia in each morphological category to total number of microglial cells in the region of interest was reported as a marker of activation state in each treatment group.

### Birefringence assay

Birefringence of skeletal muscle tissue was performed using two 82 mm circular polarizer lenses fitted to a Leica MZ16 F inverted microscope. Birefringence of zebrafish muscle tissue was performed according to previously published methods^33^. Briefly, wild type embryos exposed to BPA or vehicle control as described above were dechorionated and anaesthetized in 0.04% tricaine dissolved in E3 media and oriented flat along the lateral axis on a polarizer lens. The top lens was rotated until the axis of polarization between the two lenses was 90°. Images were acquired and focused images of the muscle tissue along the trunk were selected for analysis using ImageJ. The whole trunk of embryos was selected for analysis of the mean grey value and area of selection. Normalized values of mean grey value to area of selection were reported, error bars represent ± s.d.

### Statistics

All data analyses were performed blinded by sample coding. Data was tested for normal distribution using the Shapiro-Wilk test. Parametric data were analyzed using a student’s t-test, and non-parametric data were analyzed using Kruskal-Wallis ranked sums and Mann-Whitney U tests. Data was considered significant at P < 0.05.

## Electronic supplementary material

Supplementary Information

Video S1
