# Supplementary material for: Modeling Environmentally-Induced Motor Neuron Degeneration in Zebrafish
Source: Sci Rep. 2018 Mar 20;8:4890. doi: 10.1038/s41598-018-23018-w (PMC5861069; doi:10.1038/s41598-018-23018-w)
Supplement: Supplementary file 1 — Supplementary Information [file 41598_2018_23018_MOESM1_ESM.pdf]

Supporting Information

Modeling Environmentally-Induced Motor neuron Degeneration in Zebrafish

Jessica R Morrice[1], Cheryl Y Gregory-Evans[1-3] and Christopher A Shaw[1-3]*

[1]Department of Experimental Medicine

[2]Graduate Program in Neuroscience

[3]Department of Ophthalmology and Visual Sciences

*cashawlab@gmail.com

Supplementary data

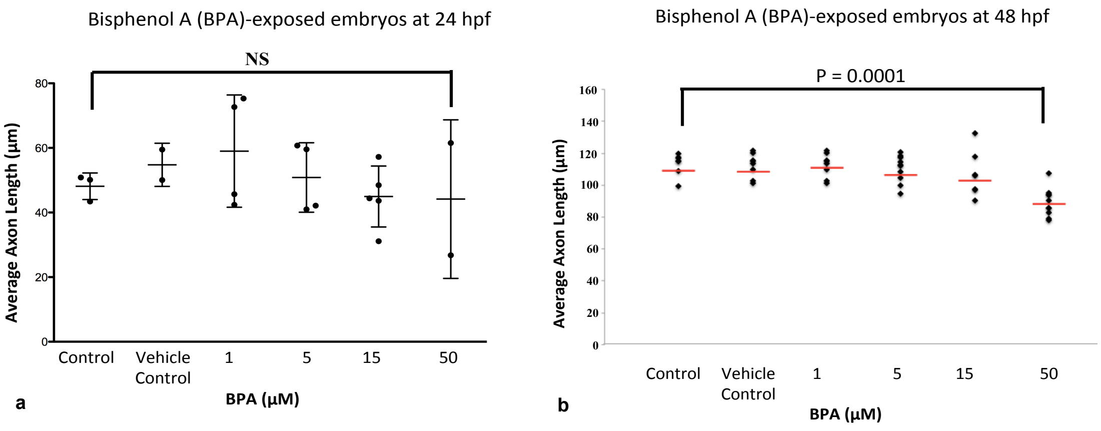

Supplementary Figure S1. Neurotoxic effect of time-dependent exposure to BPA. Wild type embryos were exposed to control, vehicle control or BPA at 6 hpf and fixed at a) 24 hpf (N= 2 – 5 biological replicates) and b) 48 hpf (P = 0.0001; N = 7-10 biological replicates). Error bars represent ± s.d., P values were determined by Mann-Whitney U test.

Supplementary Table S1. Lethality of delayed exposure at 12 hpf. Delayed exposure to BPA at 12 hpf did not increase embryonic lethality at 24 hpf or 48 hpf (% morality of total embryos). N = 11 biological replicates.

	**24 hpf**	**48 hpf**
**Vehicle Control***	0%	0%
**50 μ M BPA**	0%	0%

Embryos were subject to chronic non-static exposure starting at 12 hpf in vehicle control and treatment groups.
**1% DMSO dissolved in E3 media*
Abbreviations: BPA – Bisphenol A; hpf – hours post fertilization

Supplementary Table S2. Motor function of delayed exposure at 12 hpf. Embryos subject to delayed BPA exposure at 12 hpf reduced motor behaviour at 48 hpf using TEER (% failed motor response). N = 11 biological replicates; N = 1 technical replicate.

	**24 hpf**	**48 hpf**
**Vehicle Control*^**	0%	0%
**50 μ M BPA^**	0%	100%

Embryos were subject to chronic non-static exposure starting at 12 hpf in vehicle control and treatment groups

**1% DMSO dissolved in E3 media*

Abbreviations: BPA – Bisphenol A; hpf – hours post fertilization

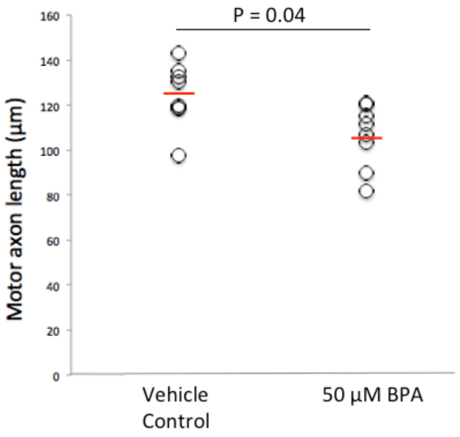

Supplementary Figure S2. The effect of neurodevelopmental exposure of BPA on motor axon length. Motor axon length was reduced in *Tg:mnx1-GFP* embryos subject to delayed exposure at 12 hpf in groups exposed to 50 μ M BPA as compared to vehicle control groups (P = 0.04, N = 8 biological replicates, N = 5 - 6 technical replicates; Mann-Whitney U test).

Supplementary Table S3. Motor Function of delayed exposure at 3 dpf. Embryos subject to delayed exposure to BPA starting at 3 dpf. Values indicate percent of *Tg:mnx1-GFP* embryos with failed motor response at 5 dpf. N = 15 biological replicates in 3 dpf, N = 8 biological replicates in 5 dpf group; N = 1 technical replicate.

**3 dpf** [Ψ]	**Exposure at 3 dpf**	**5 dpf**
6.7%	**Vehicle Control***	12.5%
	**50 μM BPA**	100%

Embryos were subject to chronic non-static exposure of treatment starting at 3 dpf in all groups.

Ψ Motor function of randomly selected embryos grown normally in embryo media was analyzed prior to either treatment exposure

**1% DMSO dissolved in E3 media*

Abbreviations: BPA – Bisphenol A; dpf – days post fertilization

Supplementary Table S4. Lethality of delayed exposure at 3 dpf. Delayed exposure to BPA at 3 dpf increased embryonic lethality at 5 dpf (% morality of total embryos). N = 120 biological replicates. Embryos subject to delayed exposure to BPA starting at 3 dpf instead of 6 hpf.

	**4 dpf**	**5 dpf**
**Vehicle Control***	0%	0%
**50 μM BPA**	3.3%	93.7%

Embryos were subject to chronic non-static exposure of treatment starting at 3 dpf in all groups.

**1% DMSO dissolved in E3 media*

Abbreviations: BPA – Bisphenol A; dpf – days post fertilization

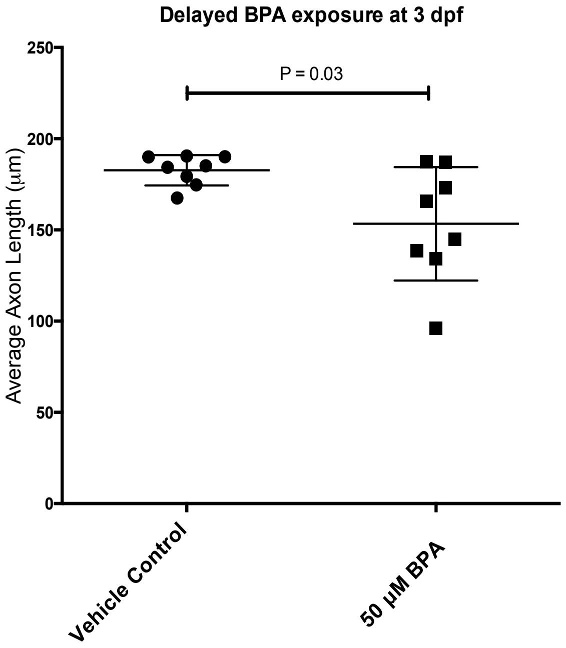

Supplementary Figure S3. Exposure to BPA at 3 dpf on motor axon length at 5 dpf. *Tg:mnx1-GFP* embryos subject to 42 hours of treatment exposure at 3 dpf instead of 6 hpf showed reduced motor axon length at 5 dpf as compared to vehicle controls. (P = 0.03, N = 8 biological replicates, N = 3 - 4 technical replicates). Error bars represent ± s.d., P value was determined by a student's t-test using Welch's correction.

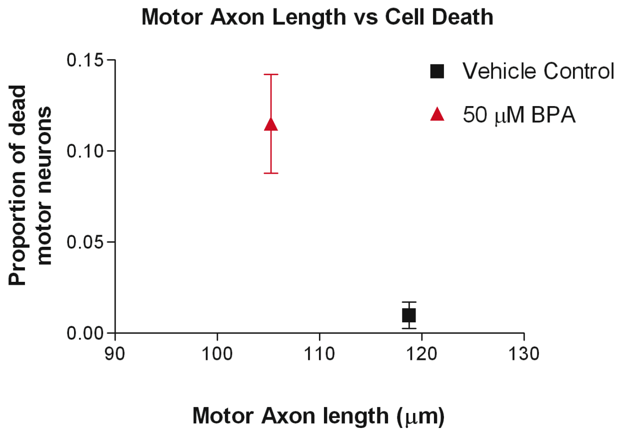

Supplementary Figure S4. Environmental toxin exposure is suggestive of retrograde motor neuron degeneration. Reduced motor axon length at 48 hpf is associated with increased motor cell death at 72 hpf in embryos exposed to 50 µM BPA. Symbol represents the group mean, error bars represent s.d.; N = 9 – 10 biological replicates.

Supplementary Table S5. Localization of activated microglia. Proportion of amoeboid microglia spatially associated with degenerating motor axons as compared to the motor cell soma in the spinal cord at 48 hpf in *Tg:mnx1-GFP/pU1-RFP* embryos (% of total microglial cells). N = 8-10 biological replicates; N = 1-15 technical replicates.

	**Microglia associated with motor axons* (%)**	**Microglia associated with soma* (%)**
**Vehicle Control**$^{\Psi}$	97	3
**50 µM BPA**	100	0

Embryos were subject to chronic non-static exposure starting at 6 hpf in vehicle control and treatment groups.

**Data are based on number of activated microglial cells spatially associated with motor axons or located in the spinal cord in 1 hemisegment within the 6 – 9 somite region.*

Ψ *1% DMSO dissolved in E3 media*

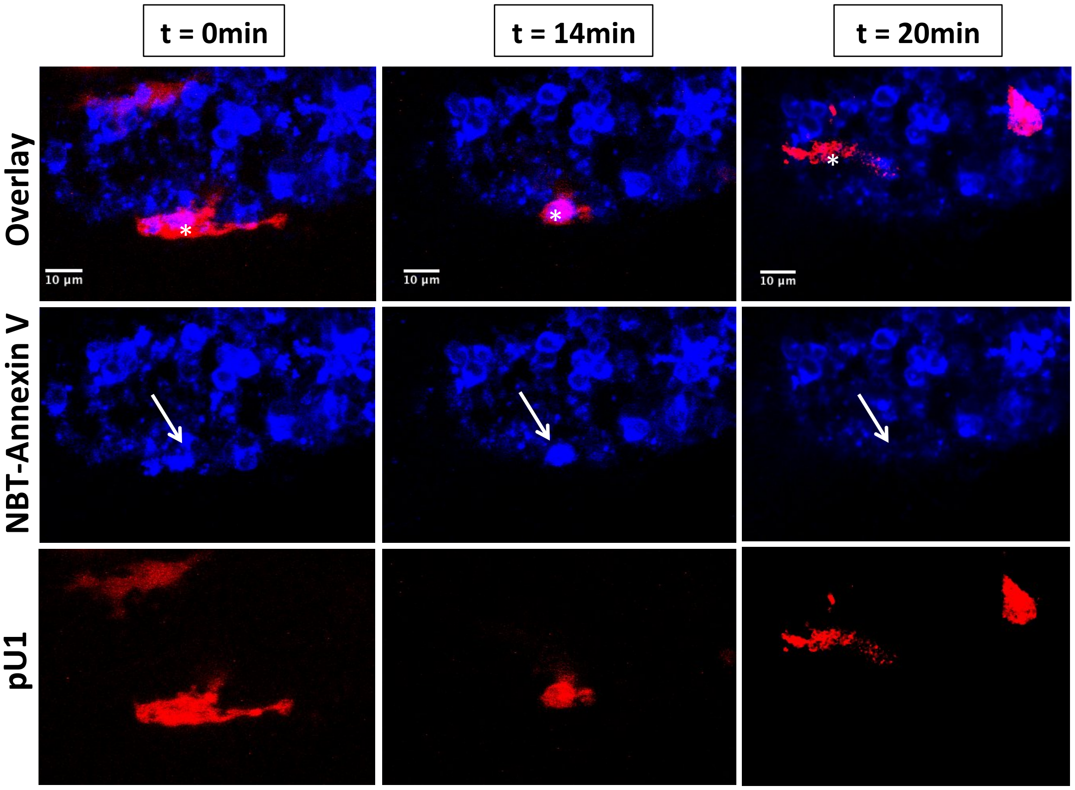

Supplementary Figure S5. Microglial engulfment of apoptotic neurons at 72 hpf. Representative static images from live imaging of a microglial cell engulfing an apoptotic neuron in the spinal cord at 72 hpf following BPA exposure in a double transgenic *Tg:pU1:Gal4-UAS-RFP/NBT:DLexPR-secA5-TagBFP* embryo, which have pU1+ microglia cells labeled in red and apoptotic neurons labeled in blue. Asterisks denotes pU1+ cell (in red) in the process of engulfing a neuron undergoing apoptosis (in blue) in the spinal cord and its subsequent migration away from this site. White arrow denotes an apoptotic neuron being removed from the spinal cord over a time course of 20 minutes. Note that specific microglia were not followed from 48 hpf to 72 hpf, and cells imaged in this figure illustrate the general microglial response in the spinal cord at 72 hpf. Images were collected from N = 1 biological replicate.

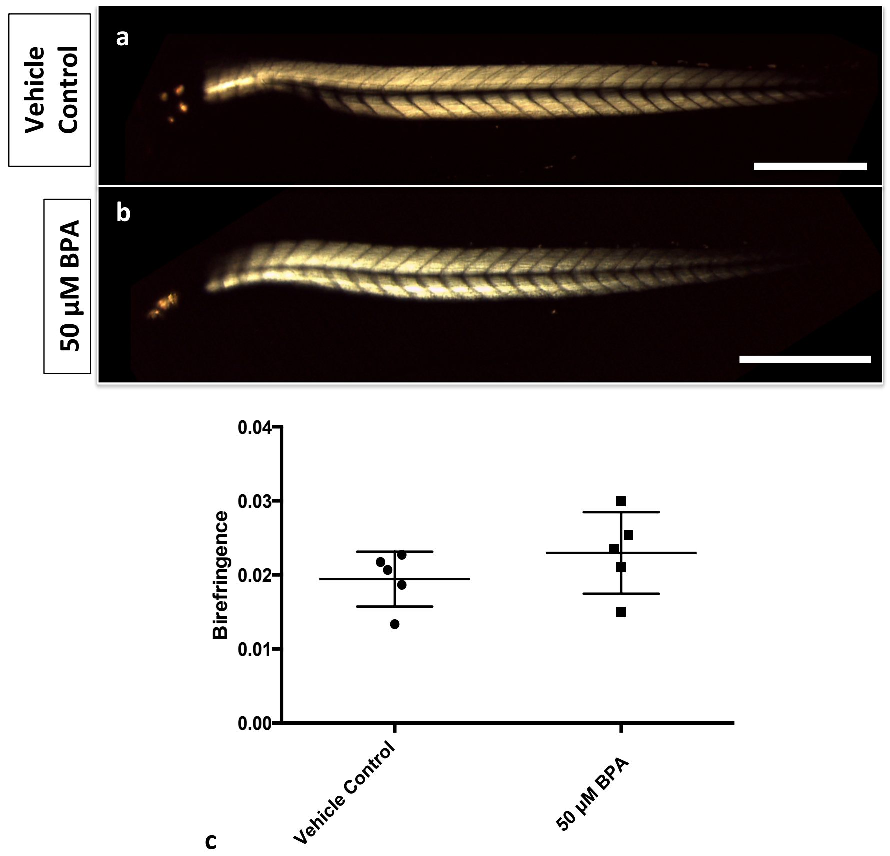

Supplementary Figure S6. Quantification of muscle birefringence (a-b) Birefringence of skeletal muscle at 48 hpf in wild type embryos exposed to vehicle control or BPA at 6 hpf. c) Wild type embryos exposed to vehicle control or 50 μM BPA had similar skeletal muscle birefringence at 48 hpf (P=0.27; N=5 biological replicates, N=1 technical replicate). Birefringence values represent the mean intensity of the total trunk normalized to the area of trunk analyzed. Scale bar = 500 µm. Data was analyzed by students t-test using Welch's correction.

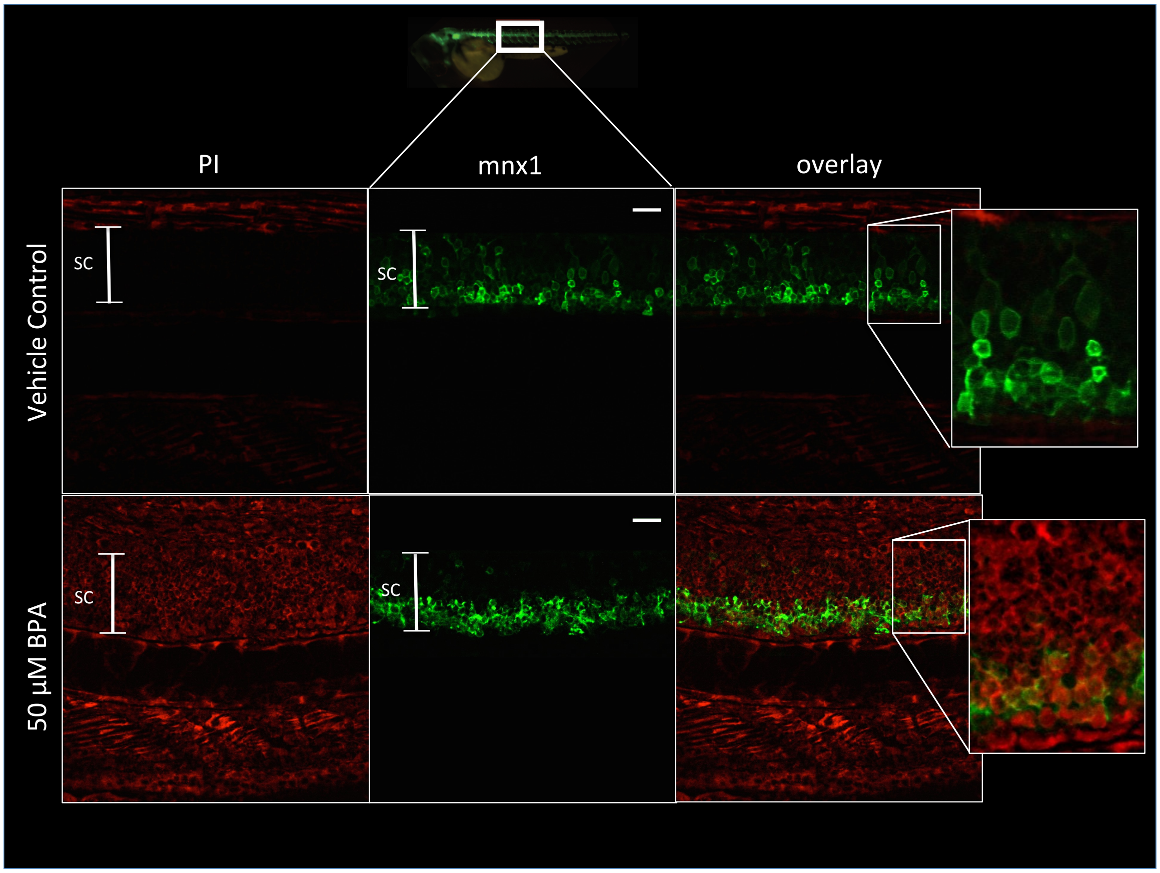

Supplementary Figure S7. Cell death caused by BPA is not specific to motor neurons. Representative images of non-specific cell death following BPA exposure. PI staining in spinal cord and musculature of embryos exposed to 50 µM BPA or vehicle control. Images represent one hemisegment within the 6 – 9 somite region of *Tg:mnx1-GFP* embryos at 3dpf. Inset images show that the neurotoxic effect of BPA is not motor neuron-specific in the spinal cord. Spinal cord (SC) is indicated by the white capped bar. Scale bar = 20 µm.

Supplementary Video S1. Activated microglia spatially interact with degenerating motor neurons. Representative 3D reconstruction of microglia interacting with motor axons in a double transgenic *Tg:mnx1-GFP/pU1-RFP* BPA-exposed embryo, which have pU1+ microglia cells shown here in magenta and motor neurons labeled in green, extending from the spinal cord. Image collected at 30 hpf by confocal microscopy and reconstructed in ImageJ. X, Y and Z axes are labeled in green. Z-stack images were collected and reconstructed from N=1 biological replicate.
